# Effect of Corneal Tilt on the Determination of Asphericity

**DOI:** 10.3390/s21227636

**Published:** 2021-11-17

**Authors:** Alejandra Consejo, Arwa Fathy, Bernardo T. Lopes, Renato Ambrósio, Ahmed Abass

**Affiliations:** 1 Department of Applied Physics, University of Zaragoza, 50009 Zaragoza, Spain; alejandra.consejo@unizar.es; 2 Institute of Physical Chemistry, Polish Academy of Sciences, 01-224 Warsaw, Poland; 3 Sixth Form, Wirral Grammar School for Girls, Bebington CH63 3AF, UK; afathy16@wirralgirls.co.uk; 4 Department of Ophthalmology, Universidade Federal de São Paulo, 1500 Vila Clementino, São Paulo 04021-001, Brazil; blopes@liverpool.ac.uk (B.T.L.); dr.renatoambrosio@gmail.com (R.A.J.); 5 Department of Civil Engineering and Industrial Design, School of Engineering, University of Liverpool, Liverpool L69 3GH, UK; 6 Department of Mechanical, Materials and Aerospace Engineering, School of Engineering, University of Liverpool, Liverpool L69 3GH, UK; 7 Department of Production Engineering and Mechanical Design, Faculty of Engineering, Port Said University, Port Said 42526, Egypt

**Keywords:** eye, cornea, tilt, asphericity, parametric, eye models

## Abstract

**Purpose:** To quantify the effect of levelling the corneal surface around the optical axis on the calculated values of corneal asphericity when conic and biconic models are used to fit the anterior corneal surface. **Methods:** This cross-sectional study starts with a mathematical simulation proving the concept of the effect that the eye’s tilt has on the corneal asphericity calculation. Spherical, conic and biconic models are considered and compared. Further, corneal asphericity is analysed in the eyes of 177 healthy participants aged 35.4 ± 15.2. The optical axis was determined using an optimization procedure via the Levenberg–Marquardt nonlinear least-squares algorithm, before fitting the corneal surface to spherical, conic and biconic models. The influence of pupil size (aperture radii of 1.5, 3.0, 4.0 and 5.0 mm) on corneal radius and asphericity was also analysed. **Results:** In computer simulations, eye tilt caused an increase in the apical radii of the surface with the increase of the tilt angle in both positive and negative directions and aperture radii in all models. Fitting the cornea to spherical models did not show a significant difference between the raw-measured corneal surfaces and the levelled surfaces for right and left eyes. When the conic models were fitted to the cornea, changes in the radii of the cornea among the raw-measured corneal surfaces’ data and levelled data were not significant; however, significant differences were recorded in the asphericity of the anterior surfaces at radii of aperture 1.5 mm (*p* < 0.01). With the biconic model, the posterior surfaces recorded significant asphericity differences at aperture radii of 1.5 mm, 3 mm, 4 mm and 5 mm (*p* = 0.01, *p* < 0.01, *p* < 0.01 & *p* < 0.01, respectively) in the nasal temporal direction of right eyes and left eyes (*p* < 0.01, *p* < 0.01, *p* < 0.01 & *p* < 0.01, respectively). In the superior–inferior direction, significant changes were only noticed at aperture radii of 1.5 mm for both right and left eyes (*p* = 0.05, *p* < 0.01). **Conclusions:** Estimation of human corneal asphericity from topography or tomography data using conic and biconic models of corneas are affected by eyes’ natural tilt. In contrast, the apical radii of the cornea are less affected. Using corneal asphericity in certain applications such as fitting contact lenses, corneal implant design, planning for refractive surgery and mathematical modelling when a geometrical centre of the eye is needed should be implemented with caution.

## 1. Introduction

During eye topography or tomography scans, patients are usually instructed to focus on a target on the topographer head placed a couple of centimetres away from their scanned eye. With the brain instructing the eye to align itself into a tilted position in order to refract light onto the foveal centre located temporal to the optic disk edge [1,2], the eye’s visual axis aligns with the topographer’s axis causing topography maps to be systematically tilted [3,4]. When using these corneal maps to generate mathematical models of the eye, it is important to consider this inherent tilt, but unfortunately, this is often not the case. Simple representations of the human eye usually ignore the effect of the hemispheric division of the brain on the eye position [5], and consequently, the outcomes of such representations do not accurately reflect the eye’s behaviour [6,7].

Over the past decades, many different methods for mathematically modelling the eye parametrically have been developed [8]. However, the accuracy of the human eye anatomic shape measurements in vivo is becoming increasingly accurate due to current advances in eye-scanning technology, hence creating the need to estimate the accuracy of these modelling approaches [9]. Using the perfect spherical model (Equation (1)) prevented the peripheral cornea from being representative enough, and so a conic model (Equation (2)) was introduced to eliminate the consequences of spherical aberration [8]. The curvature of the eye surface was controlled using an asphericity coefficient $\left(Q\right)$ where when Q<0, the surface becomes flatter in the direction of the periphery. Therefore, corneal asphericity measures how much a corneal surface deviates from its perfect spherical equivalent shape:

(1) $$z\;\left(x,y\right)=\sqrt{R^{2}-\left(x^{2}+y^{2}\right)}-R$$

where R denotes the radius of the spherical model, later referred to as apical radius, which is a term often used in optometry and ophthalmology to indicate the curvature at the corneal apex, and

(2) $$z\;\left(x,y\right)=\frac{\sqrt{R^{2}-\left(x^{2}+y^{2}\right)\left(Q+1\right)}-R}{Q+1}$$

where R denotes the radius of the conic model, and Q is the asphericity coefficient.

Subsequentially, and for simplicity, a shape factor (κ) was used in some studies [10,11] defined as

(3) κ=Q+1

where a perfectly spherical surface is attained when κ is set to one (Q=0), and a flatter peripheral surface can be achieved when κ is set to a value lower than one (Q<0).

Notably, the effects caused by astigmatism are ignored in the conic model, which assumes rotational symmetry. With-the-rule astigmatism results in flatter curvature at the nasal–temporal meridian than the superior–inferior corneal meridian, while against-the-rule astigmatism causes an opposite effect and oblique astigmatism is identified when the principal meridians are not at 90° and 180°. This phenomenon is known as toricity [12,13,14], and when added to the conic model, it forms biconic surfaces [15]. The change concerning the already described conic model that forms the biconic model (Equation (4)) consists of adding two parameters, $R_{x}$ and $R_{y}$, to represent corneal radii in the principal directions and two asphericity coefficients, $Q_{x}$ and $Q_{x}$, to control the steepness of the eye’s surfaces in two perpendicular directions.

(4) $$z\;\left(x,y\right)=\frac{-\left(x^{2}R_{x}{}^{-1}+y^{2}R_{y}{}^{-1}\right)}{1+\sqrt{1-\left(1+Q_{x}\right)R_{x}{}^{-2}x^{2}-\left(1+Q_{y}\right)R_{y}{}^{-2}y^{2}}}$$

Biconic models can be rotated around the Z-axis by an angle $\alpha_{z}$ to further align the principal directions to the astigmatism axis to construct eye models using topography data [4,8].

Values for corneal conic asphericity, Q, have ranged down to −0.82 in the literature [16,17,18,19,20,21,22,23,24,25,26,27,28,29,30,31,32,33]. On the other hand, biconic asphericity recorded values range down to −0.28 [33,34], contingent on the sample size and the fitting algorithm used. For example, Ying produced a conic model with asphericity coefficients varying from 0.18 to 0.3 due to astigmatism-skewed distribution [35], that provided a corneal radius of 7.83 mm [12]. When tangential corneal radius was utilized instead of sagittal radius curvature, the asphericity coefficient Q varied from −0.33 to 0.12 between principal corneal meridians [36].

This cross-sectional study was designed to quantify the effect of levelling the corneal surface around the optical axis on the calculated values of corneal asphericity when conic and biconic models are used to fit the anterior corneal surface. The study uses a newly developed, already validated method that allows the determination of the corneal optic axis from corneal topography [37] to level the corneal surfaces around its optic axis, and then quantifies and compares the corneal asphericity calculated from the raw and the levelled corneal surface.

## 2. Materials and Methodology

### 2.1. Mathematical Simulation

**Figure 1:**
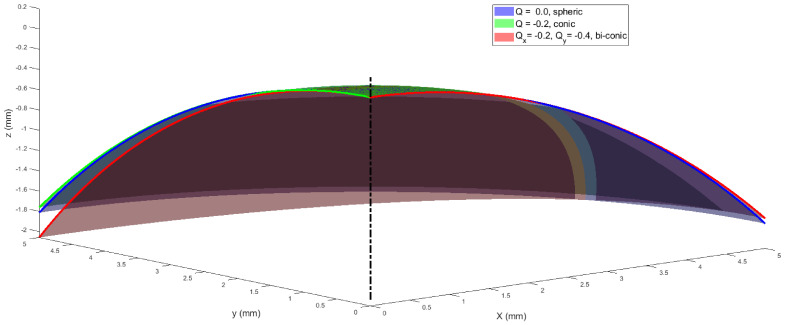
Simulated spheric, conic and biconic surfaces with a nominal radius of 7.8 mm in all cases, except in the biconic model, where Ry was set to 7 mm while Rx was kept to 7.8 mm. In this example, radius of aperture was set to 5.0 mm.

The effects of tilting spherical, conic and biconic surfaces were investigated by simulating the corresponding parametrical models. The study was carried out as a proof of concept before clinical data analysis. In this simulation, three surfaces (spheric, conic and biconic, Figure 1) were generated over regular grids covering radii of apertures (r) of 1.5 mm, 3.0 mm, 4.0 mm and 5.0 mm, where the radius of aperture simulates the iris size. The apical radius of the spherical surface was used as a control variable, since, by definition, it is a fixed parameter. This is because the apical radius of a perfectly spherical surface is not affected by rotation. A nominal apical radius of 7.8 mm, which represents the average corneal apical radius [38], was used to construct all three models, except the biconic model, where R_y_ was set to 7 mm to represent astigmatism while R_x_ was kept to 7.8 mm [27]. The asphericity Q was set to zero in the spherical model, -0.2 in the conic model and Q_x_ = −0.2, Q_y_ = −0.1 in both X and Y directions in the biconic model. Tilt was introduced by rotating the surface around the Y-axis by an angle α_y_ that represents the magnitude of the rotation in a 3D Euclidean space, then shifting the apex to the origin position (0,0,0). At the same time, the surface is located on the negative Z-axis side to mimic the way topography data is usually presented, as seen in Figure 1 [39]. Surface rotations were accomplished through three-dimensional rotation matrices [40], where both angles of rotation around the X-axis and the Y-axis α_x_, α_y_ were set to zero.

### 2.2. Clinical Data Collection and Processing

The current study utilises fully anonymised records from 177 participants aged 35.4 ± 15.2, retrospectively evaluated in solely secondary analyses. Participants are healthy subjects selected from referrals to Instituto de Olhos Renato Ambrósio (Rio de Janeiro, Brazil) from January 2010 to December 2014. No clinical data were collected specifically for this study; therefore, no ethical approval was required according to the policy of the University of Liverpool on research ethics. Nevertheless, the study was conducted in accordance with the standards set in the Declaration of Helsinki. The participants’ corneas were measured using the Pentacam HR system, and the values obtained were the following: flat curvature in the central 3 mm zone (K_1_) of 42.6 ± 1.4 D, steep curvature in the central 3 mm zone (K_2_) of 43.8 ± 1.5 D, and mean curvature in the central 3 mm zone (Km) of 43.2 ± 1.4 D.

Clinical topography data were collected from both right and left eyes of healthy participants with no history of ocular disease, trauma or ocular surgery using the Pentacam HR (OCULUS Optikgeräte GmbH, Wetzlar, Germany) before being anonymized. Those who wore soft contact lenses less than 2 weeks before measurement or rigid gas permeable (RGP) contact lenses less than 4 weeks before measurements were excluded, as well as those with intraocular pressure (IOP), measured by the Goldmann Applanation Tonometer, higher than 21 mmHg. The Pentacam raw elevation data was exported in CSV format and analysed using custom-built MATLAB (MathWorks, Natick, MA, USA) code.

### 2.3. Determination of the Optical Axis from Clinical Data

The corneal optic axis is defined in the literature as that path of light that goes through the ocular system without refraction [41]. In this work, an already validated methodology to assess the corneal optical axis was used [37]. To determine the corneal optical axis, a light ray-tracing algorithm was coded in MATLAB software and graphically validated using AutoCAD software (Autodesk, McInnis Parkway San Rafael, CA, USA). This was achieved by simulating parallel light rays to pass and refract through the cornea’s anterior and posterior surfaces in accordance with Snell’s law [12,42]. The angle of incidence for the light rays in the air was calculated as the angle between the ray and the normal to the corneal surface. The direction of the refracted ray was calculated while it passed through corneal depth with the refractive indices of air, cornea, and aqueous set to 1.0, 1.376 and 1.336, respectively, following Gullstrand’s relaxed eye model [42,43]. The light was then refracted again at the cornea’s posterior surface, with the previous angle of refraction used as the angle of incidence, before being refracted once more through the aqueous.

The next task involved locating the intersection point between the refracted light ray and the corneal longitudinal axis. The optical power was estimated using the distance between this intersection point and the corneal apex [44]. It was found during this analysis that not all rays intersected the corneal axis due to spherical aberration. This meant that the closest point to the corneal visual axis was used as the focal point for these rays [45].

With the optical axis defined as a straight light ray that enters and leaves an optical system along the same line [46] without refraction, the path for that light ray may be located between two points on the corneal anterior and posterior surfaces. When a light ray passes through both corneal surfaces without being refracted, the focal length will be infinity, and its power will tend towards zero. Each eye’s corneal topography data was put in an optimization loop where it was rotated in three dimensions. At the same time, the simulated light rays are kept parallel towards the anterior corneal surface, in accordance with previous work [37].

The loop was set in such a way that when one of the light rays recorded an infinite focal length and therefore an optical power of zero, the optimization procedure would end. This was achieved using the Levenberg–Marquardt nonlinear least-squares algorithm (LMA) [47,48] in MATLAB’s Optimisation Toolbox, where the algorithm was set to end when the smallest ray’s optical power was below $10^{-20}$ D. The corneal surfaces were rotated around the X-axis and Y-axis by angles $\alpha_{x}$ and $\alpha_{y}$, respectively, to minimize the optical power and then use it as the optimal optical axis. This process produced optimum values for rotation angles $\alpha_{x}$ and $\alpha_{y}$, which can be made use of to locate the best location for the optical axis. The rotation was accomplished by the following three rotation matrices [40], where $\alpha_{z}$ was set to zero.

(5) $$R_{x}\left(\alpha_{x}\right)=\left[\begin{array}{ccc} 1 & 0 & 0\\ 0 & \cos\alpha_{x} & -\sin\alpha_{x}\\ 0 & \sin\alpha_{x} & \cos\alpha_{x} \end{array}\right]$$

(6) $$R_{y}\left(\alpha_{y}\right)=\left[\begin{array}{ccc} \cos\alpha_{y} & 0 & \sin\alpha_{y}\\ 0 & 1 & 0\\ -\sin\alpha_{y} & 0 & \cos\alpha_{y} \end{array}\right]$$

(7) $$R_{z}\left(\alpha_{z}\right)=\left[\begin{array}{ccc} \cos\alpha_{z} & -\sin\alpha_{z} & 0\\ \sin\alpha_{z} & \cos\alpha_{z} & 0\\ 0 & 0 & 1 \end{array}\right]=\left[\begin{array}{ccc} 1 & 0 & 0\\ 0 & 1 & 0\\ 0 & 0 & 1 \end{array}\right]$$

Following the elemental rotation rule, the rotated coordinates of the corneal surface $X_{r}$, $Y_{r}$ and $Z_{r}$ were calculated as:

(8) $$\left[\begin{array}{ccc} x_{r1} & x_{r2} & x_{r3}\\ y_{r1} & y_{r2} & y_{r3}\\ z_{r1} & z_{r2} & z_{r3} \end{array}\begin{array}{ccc} & \ldots & x_{\mathrm{rn}}\\ & \ldots & y_{\mathrm{rn}}\\ & \ldots & z_{\mathrm{rn}} \end{array}\right]=\left[R_{x}\left(\alpha_{x}\right){*R}_{y}\left(\alpha_{y}\right){*R}_{z}\left(\alpha_{z}\right)\right]*\left[\begin{array}{ccc} x_{1} & x_{2} & x_{3}\\ y_{1} & y_{2} & y_{3}\\ z_{1} & z_{2} & z_{3} \end{array}\begin{array}{ccc} & \ldots & x_{n}\\ & \ldots & y_{n}\\ & \ldots & z_{n} \end{array}\right]$$

The light ray-tracing process then continued in the optimization loop after each rotation. The process was set to stop when the smallest ray’s optical power was below 1 × 10^−20^ D.

### 2.4. Fitting the Corneal Surface to Spherical, Conic and Biconic Models

With asphericity varying based on the aperture radius, a set of radii (r_max_) of 1.5, 3.0, 4.0 and 5.0 mm were used in the fitting exercise for the spherical, conic and biconic models. Surface data for each aperture radius was considered one-by-one, with data beyond each radius set to NaN (‘Not a Number’ in MATLAB) to be disregarded. This allows the surface grid to be centred around the corneal apex with the radius of each point $R_{g}$ calculated as

(9) $$R_{g}=\sqrt{X_{g}{}^{2}+Y_{g}{}^{2}}Z_{g}\left(R_{g}>r_{\max}\right)=\mathrm{NaN}$$

where $X_{g}$ and $Y_{g}$ represent the grid points in the nasal–temporal and superior–inferior directions, respectively, and $Z_{g}$ is the corneal raw elevation. Once the surface data within the r_max_ aperture radius had been identified, the fitting was completed by minimizing the fitting error (Err), as shown in Equation (10),

(10) $$\mathrm{Err}=\frac{1}{k}\overset{k}{\underset{i=1}{\sum }}{\left(Z_{i_{\mathrm{fit}}}-Z_{i_{\mathrm{surf}}}\right)}^{2}$$

where $Z_{i_{\mathrm{fit}}}$ is the fitted surface, $Z_{i_{\mathrm{surf}}}$ is the measured raw elevation surface height and k is the number of data points. This minimization process was also carried out by the LMA [47,48] via the MATLAB Optimisation Toolbox. The spherical model outputted one apical radius, R, per aperture per cornea, the conic model resulted in one apical radius, R, and a single asphericity value, Q, per aperture per cornea. Lastly, the biconic model resulted in two apical radii R_x_, R_y_ and two asphericity values Q_x_, Q_y_ per aperture per cornea.

### 2.5. Statistical Analysis

Statistical analysis was conducted using MATLAB’s Statistics and Machine Learning Toolbox (MathWorks, Natick, MA, USA). The null hypothesis probability (*p*) was calculated at a 95% confidence level. One-sample Kolmogorov–Smirnov test was applied on each investigated dataset to ensure that it followed normal distribution [49,50,51]. Paired sample *t*-tests were used to examine the significance between samples of datasets in order to determine whether the results represent an independent record.

## 3. Results

This section states the core findings of the two aspects of the current study; simulation-based results and clinical-based results built upon the qualitative research methodology introduced in the Methods section.

### 3.1. Simulation-Based Results

**Figure 2:**
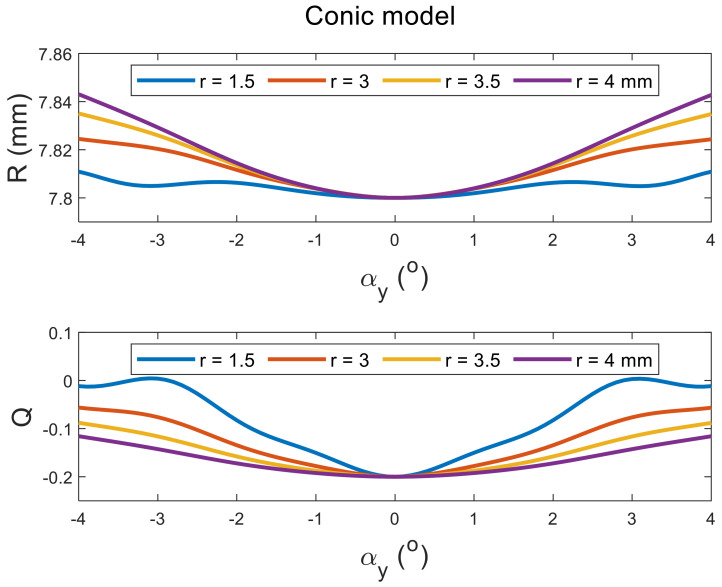
Effect of tilting a simulated conic surface of nominal apical radius of 7.8 mm and nominal asphericity of −0.2 on its radius and asphericity as fitted in tilted positions at different aperture sizes. Tilt has been introduced by rotating the surface around the Y-axis, then shifting the apex to the origin position.

**Figure 3:**
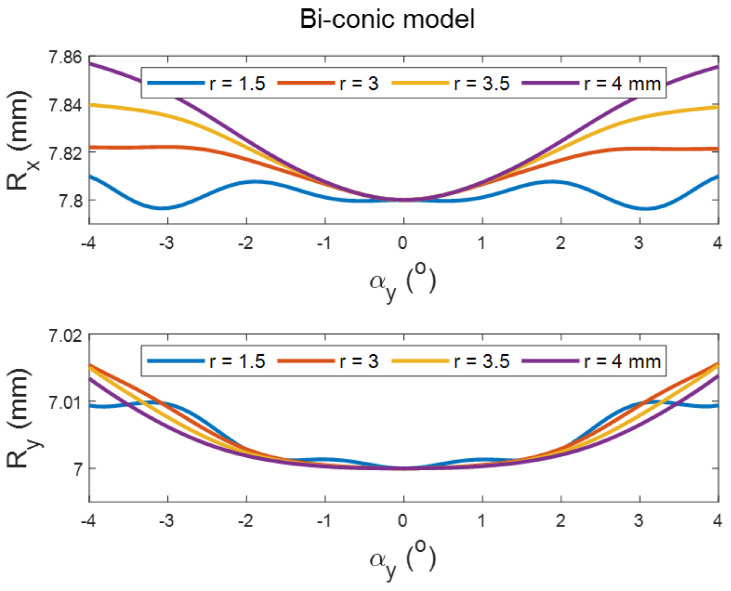
Effect of tilting a simulated biconic surface of apical radii R_x_ = 7.8 mm, R_y_ = 7 mm and asphericities of Q_x_ = −0.2, Q_y_ = −0.1 on its radius as fitted in tilted positions. Tilt has been introduced by rotating the surface around the Y-axis, then shifting the apex to the origin position.

**Figure 4:**
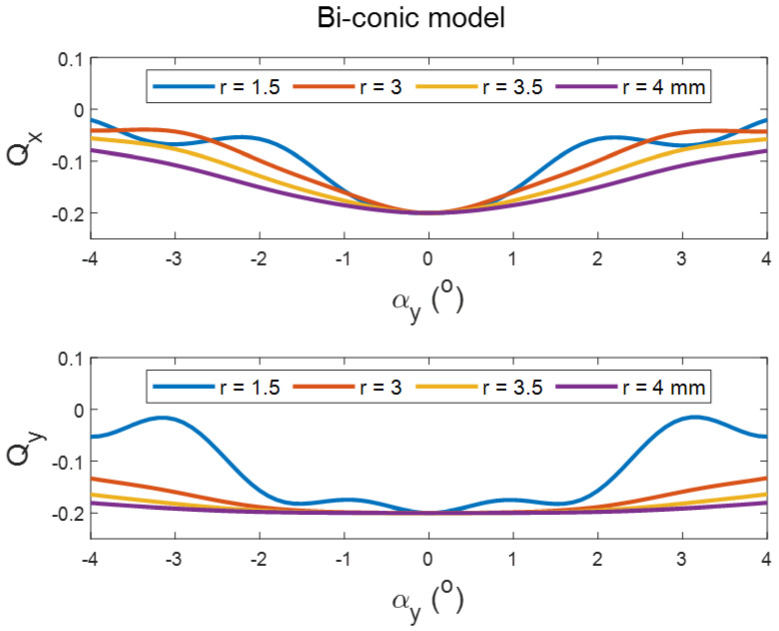
Effect of tilting a simulated biconic surface of apical radii R_x_ = 7.8 mm, R_y_ = 7 mm and nominal asphericities of Q_x_ = −0.2, Q_y_ = −0.1 on its asphericity as fitted in tilted positions. Tilt has been introduced by rotating the surface around the *Y*-axis, then shifting the apex to the origin position.

Tilting the perfect spherical surface did not cause any change in its apical radius, as expected. However, it did cause an increase in the apical radii of the surface with the increase in the tilt angle in both positive and negative directions, and aperture radii in conic and biconic models (Figure 2 and Figure 3, respectively). Different effects were noticed when the asphericity was investigated, as the value of Q tends to increase with the tilt angle but decreases with the increase in the aperture radii (see Figure 2 and Figure 4). It can be seen from the simulation results that the tilt effect of aperture radius r = 1.5 mm was quite different from other aperture radii, as there was a wavy shape in response to the tilt in both radii of the surface and asphericities.

### 3.2. Clinical-Based Results

**Figure 5:**
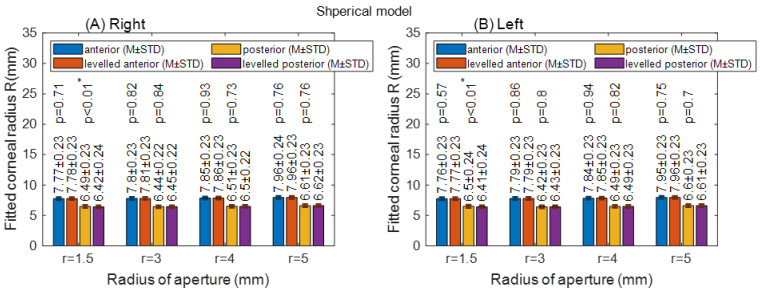
Corneal apical radius (R) fitted to spherical model with different radii of aperture (r). The asterisk (*) indicates the statical significance (**A**) Right corneas; (**B**) Left corneas.

Fitting the corneal to spherical models did not show a significant difference between the raw-measured corneal surfaces and the levelled surfaces for right and left eyes, except at a radius of aperture 1.5 mm for the anterior corneal surface (*p* < 0.01), Figure 5. There was no asphericity to report with spherical models, as it must be set to zero by definition in all cases.

**Figure 6:**
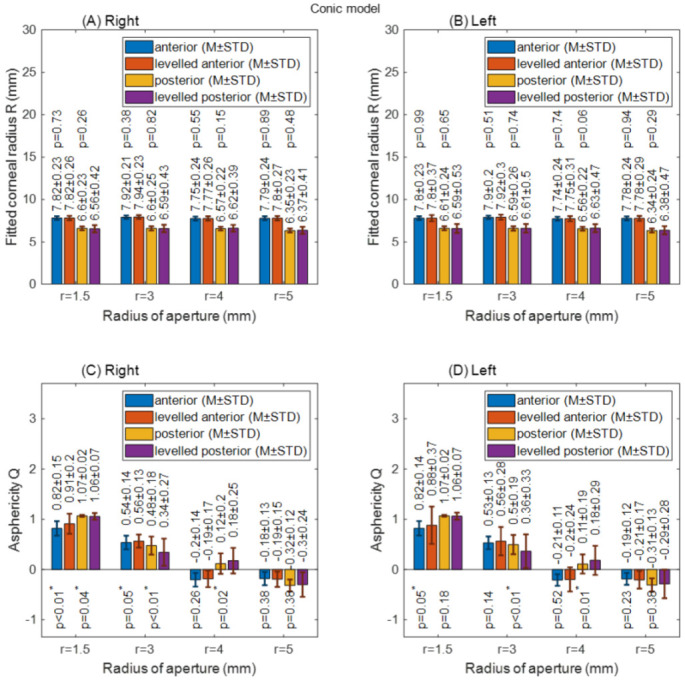
Corneal apical radius (R) and asphericity (Q) fitted to conic model with different radii of aperture (r). (**A**) Right corneal fitted radii; (**B**) Left corneal fitted radii; (**C**) Right corneal asphericities; (**D**) Left corneal asphericities.

When the conic models were fitted to the cornea, changes in the radii of the cornea among the raw-measured corneal surfaces’ data and levelled data were not significant; however, statistically significant differences were recorded in the asphericity of the anterior surfaces at radii of aperture 1.5 mm (*p* < 0.01) and 3 mm (*p* = 0.05) in right eyes, and at a radius of aperture 1.5 mm (*p* = 0.05) in left eyes. The posterior surface asphericity recorded a significant change among raw-measured data and levelled data at an aperture radius of 3 mm (*p* = 0.02 for left eyes and *p* = 0.01 for right eyes), as seen in Figure 6.

**Figure 7:**
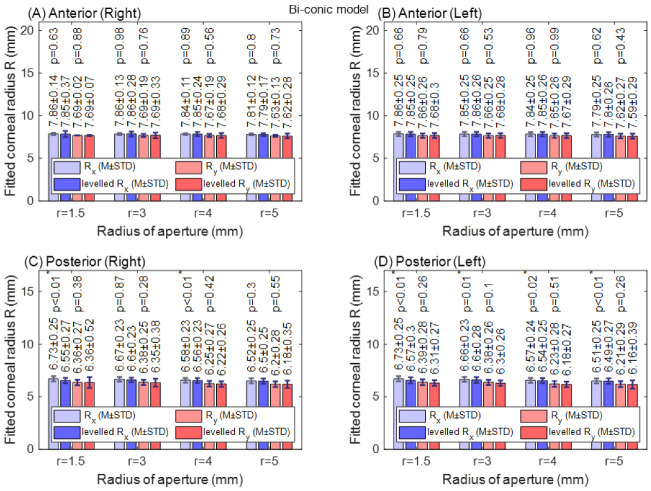
Corneal apical radius (R) fitted to biconic model with different radii of aperture (r). R_x_: nasal–temporal. R_y_: superior–inferior. The asterisk (*) indicates the statical significance. (**A**)Anterior right corneal fitted radii; (**B**) Anterior left corneal fitted radii; (**C**) Posterior right corneal fitted radii; (**D**) Posterior left corneal fitted radii.

**Figure 8:**
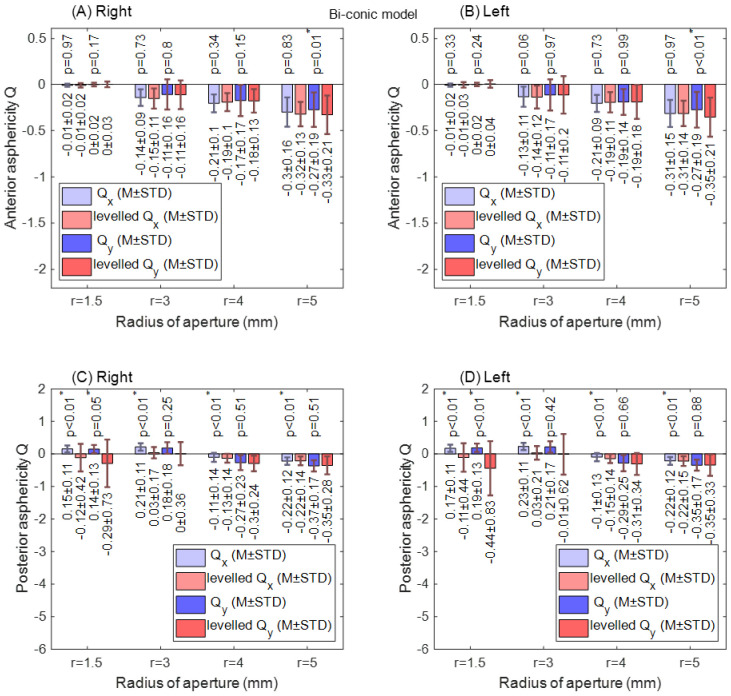
Corneal asphericity (Q) fitted to biconic model with different radii of aperture (r). The asterisk (*) indicates the statical significance. (**A**)Anterior right corneal asphericities; (**B**) Anterior left corneal asphericities; (**C**) Posterior right corneal asphericities; (**D**) Posterior left corneal asphericities.

With the biconic model, no significant difference in corneal radii was found among raw-measured and levelled Rx and Ry data in both right and left eye anterior surfaces for all radii of apertures, as shown in Figure 7. When the anterior surface asphericity was investigated in both the nasal–temporal (R_x_) direction and the superior–inferior (R_y_) direction, the only significant difference was noticed at an aperture radius of 5 mm in the superior–inferior direction. This has been noticed in both right and left eyes, as seen in Figure 8A,B. Unlike the anterior surface, the posterior surface recorded significant differences at aperture radii of 1.5 mm, 3.0 mm, 4.0 mm and 5.0 mm (*p* = 0.01, *p* < 0.01, *p* < 0.01 & *p* < 0.01, respectively) in the nasal–temporal direction of right eyes and left eyes (*p* < 0.01, *p* < 0.01, *p* < 0.01 & *p* < 0.01, respectively), as shown in Figure 8C,D. In the superior–inferior direction, significant changes were noticed at aperture radii of 1.5 mm only for both right and left eyes (*p* = 0.05, *p* < 0.01).

## 4. Discussion

The theoretical investigation showed that the asphericity of tilted conic or biconic surfaces is affected by the tilt in these surfaces, which indicated that determining the asphericity of corneas from their raw scanned data could be misleading. The cause of this is that scanned topography or tomography data of the eye is usually tilted as a result of fixation on a close target during the eye surface scan. Both asphericity and the apical radius of the cornea should be calculated with respect to the geometrical centre of the cornea, but the current common practice is that they are computed from raw measured topography or tomography data; therefore, they are calculated with respect to the corneal visual axis, which is not identical to the eye’s optical axis and cannot be considered as a geometrical axis.

Corneal asphericity has been linked to myopia [52,53], refractive error [29,54,55], spherical aberration [56], binocular summation [22], laser in situ keratomileusis (LASIK) [31,57,58], retinal image quality [24], intrastromal corneal ring segments (ICRS) [19], contact lenses [59] and ethnicity [60]. It was also deemed to be not useful in predicting the refractive outcome of radial keratotomy [61] when determined from topography data.

The current study results agree with Douthwaite’s results [62] that apical radii are slightly affected by corneal tilt when fitted to conic models, and also agrees that this is not the case with the corneal asphericity [63], as significant changes in the corneal asphericity were observed up to aperture radius of 4 mm. The only exceptions to this significance were noticed in the posterior surfaces of the left eyes at aperture radii of 1.5 mm and 3.0 mm, and this is likely to be linked to two elements. Firstly, the wavy nature of the change in the asphericity with the tilt angle that has been observed in the mathematical simulation, Figure 2, Figure 3 and Figure 4. Secondly, right eyes tend to tilt more than left eyes during the fixation on near targets [4], as two-thirds of the population is believed to be right-eye dominant [64,65,66,67,68]. Other factors cause some differences among fellow eyes, such as variation in the vision field among right eyes and left eyes [69], and fact that the image merging processes carried out within the brain for the two eyes are distinct [70,71].

Horizontal and vertical corneal apical radii fitted to the biconic model with different radii of aperture did not record any significant changes between levelled and raw-measured surfaces in the anterior surfaces for both fellow eyes; however, posterior surfaces showed some significant changes in the horizontal direction only. This is expected to be because of the nature of the tilt, as it is usually in the nasal–temporal direction more than the superior–inferior direction because the foveal centre is located around 2.5 mm temporal to the optical axis and slightly inferior [2]. The study also revealed that the changes in the asphericity among raw-measured corneal surfaces and levelled surfaces in the nasal–temporal direction are insignificant; however, significant changes were noticed in posterior surfaces in both nasal–temporal and superior–inferior directions. This indicates that the aspherity of the posterior corneal surface is more sensitive to the corneal tilt than the anterior surface. This finding is supported by Dubbelman, who reported no correlation between the asphericity of anterior and posterior corneal surfaces [27]. This is anticipated to be because the rate of flattening of the posterior corneal surface is more than the rate of flattening along the averaged anterior corneal surface [72].

The study has some limitations, though. The clinical data gathered for this study are collected from a single population, while there are topographical and anatomical differences between ethnic groups [73,74] that might not be reflected in this study. It is also important to point to studies that have suggested that, on the one hand, the repeatability of Scheimpflug devices is lower for the posterior corneal surface than for the anterior surface [75,76]; however, on the other hand, measurements taken with the Pentacam are described as repeatable and reproducible when they are obtained with the HR settings [77].

While the current study suggests that the estimation of corneal asphericities from topography or tomography data using conic and biconic models of the human corneas are expected to be affected by eyes’ tilt, the apical radii of the cornea are less affected by this tilt. This indicates that regarding corneal asphericity in certain applications, such as fitting contact lenses, corneal implant design, refractive surgery planning and mathematical modelling, when a geometrical centre of the eye is needed, this should be conducted with caution, as the eye is aligned with its visual axis during the scanning process, not its optical axis. Future studies to investigate the impact of the corneal tilt in these clinical applications are being conducted by our research group.

## Data Availability

Not applicable.
